# Structural Analysis and Biological Toxicity of Aflatoxins B1 and B2 Degradation Products Following Detoxification by *Ocimum basilicum* and *Cassia fistula* Aqueous Extracts

**DOI:** 10.3389/fmicb.2016.01105

**Published:** 2016-07-14

**Authors:** Wajiha Iram, Tehmina Anjum, Mazhar Iqbal, Abdul Ghaffar, Mateen Abbas, Abdul Muqeet Khan

**Affiliations:** ^1^Institute of Agricultural Sciences, University of the PunjabLahore, Pakistan; ^2^Health Biotechnology Division, National Institute for Biotechnology and Genetic EngineeringFaisalabad, Pakistan; ^3^Department of Chemistry, University of Engineering and TechnologyLahore, Pakistan; ^4^Quality Operating Laboratory, University of Veterinary and Animal SciencesLahore, Pakistan

**Keywords:** plant extract, aflatoxin, degradation, LCMS/MS, toxicity

## Abstract

This study showed the comparison between *Ocimum basilicum* and *Cassia fistula* (leaves and branch) aqueous extracts for their ability to detoxify of aflatoxins B1 and B2 (AFB1; 100 μg L^-1^ and AFB2; 50 μg L^-1^) by *In Vitro* assays and decontamination studies. Results indicated that *O. basilicum* leaves extract was found to be highly significant (*P* < 0.05) in degrading AFB1 and AFB2, i.e., 90.4 and 88.6%, respectively. However, *O. basilicum* branch, *C. fistula* leaves and branch extracts proved to be less efficient in degrading these aflatoxins, under optimized conditions, i.e., pH 8, temperature 30°C and incubation period of 72 h. Moreover the antifungal activity of these plants extracts were also tested. The findings depicted that *O. basilicum* leaves extract showed maximum growth inhibition of aflatoxigenic isolates, i.e., 82–87% as compared to other tested plants extracts. The structural elucidation of degraded toxin products by LCMS/MS analysis showed that nine degraded products of AFB1 and AFB2 were formed. MS/MS spectra showed that most of the products were formed by the removal of double bond in the terminal furan ring and modification of lactone group indicating less toxicity as compared to parent compounds. Brine shrimps bioassay further confirmed the low toxicity of degraded products, showing that *O. basilicum* leaves extract can be used as an effective tool for the detoxification of aflatoxins.

## Introduction

Mycotoxins are chemically and biologically active secondary metabolites (of molecular weight ≤ 700), produced by filamentous fungi that readily colonize crops in the field or after harvest (Richard, 2007; Turner et al., 2010). Currently, more than 400 compounds are recognized as mycotoxins, among them aflatoxins have assumed significance due to their deleterious effects on humans, poultry, and livestock (Sur and Celik, 2003; Wild and Montesano, 2009). Aflatoxins are group of heterocyclic, oxygen containing mycotoxins that possess the bisdifuran ring system. About 18 different types of aflatoxins are identified among them most commonly occurring ones are aflatoxins B1, B2, G1, G2, M1, and M2 which are mostly produced by *Aspergillus flavus* and *A. parasiticus*. Aflatoxins were first identified as the portable toxin that destroyed more than 100,000 turkey poults (turkey X disease) in England in the early 1960s (Kuhn and Ghannoum, 2003). In Kenya, (April 2004) an outbreak of jaundice with a high fatality rate was reported in humans due to aflatoxin poisoning from eating contaminated home grown maize, and resulted in 317 cases and 125 deaths (Baumgartner et al., 2005). There are strict regulations limiting mycotoxins levels in 77 countries, with specific regulatory levels for aflatoxins in food and feed stuffs (Bhatnagar et al., 2006). This shows that contamination of agricultural commodities with aflatoxin has been the subject of serious concern on an international level. Such contamination occurs by the invasion of aflatoxigenic strains ubiquitously found before and during harvesting, or by improper storage of agricultural commodities (Hwan and Choi, 2007). The food and agriculture organization (FAO) estimates that about 1000 million metric tons of foodstuff could be contaminated with mycotoxins each year (Bhat et al., 2010).

In recent years, the need to develop disease control measure as alternative to chemicals has been a priority of scientists worldwide (Reddy et al., 2009). Therefore it is important to find a practical, cost effective, and non-toxic method to prevent fungal and mycotoxin contamination in foods and feeds. Natural plant products are of interest as a source of safer or more effective substitutes for synthetic antimicrobial agents and may provide alternative way to prevent food or feed from fungal or mycotoxin contamination. Large-scale application of plant products have attracted the attention of several microbiologists as they are biodegradable, biologically safe, cost effective, renewable in nature and at the same time, conveniently used as eco-friendly technique to detoxify mycotoxins (Varma and Dubey, 1999). Previously documented literature showed that powder and extracts of various herbs and medicinal plants found to be effective in detoxifying aflatoxins and growth inhibition of toxigenic fungi. Krishnamurthy and Shashikala (2006) worked on inhibition of aflatoxin B1 production by *Aspergillus flavus*, isolated from soybean seeds by incubation with certain natural plant products at different time interval. The outcome of their study showed that captan, neem cake, leaf powder of *Withania somnifera* (Linn.), peel powder of *Camellia sinensis* (Linn.), *Citrus medica* (Linn.), and pongamia cake controlled the aflatoxin B1 production. They concluded that all the natural product treatments applied were significantly effective in inhibiting aflatoxin B1 production. In a more comprehensive study in Satish et al. (2007) scrutinized aqueous extract of 52 plants for their antifungal potential against eight important species of *Aspergillus*. They observed that among tested plants, aqueous extracts of *Acacia nilotica* (Linn.), *Eucalyptus globules* Labill, *Achras zapota* (Linn.), *Datura stramonium* (Linn.), *Emblica officinalis* (*Gaertn.*), *Lawsonia inermis* L. (henna), *Mimusops elengi* (Linn.), *Peltophorum pterocarpum* (DC.) Baker ex. K. Heyne*, Polyalthia longifolia* Benth. and Hook, *Prosopis juliflora* (Sw.) DC, *Punica granatum* (Linn.), and *Syzygium cumini* (Linn.) exhibited significant antifungal activity against *Aspergillus* species. Correspondingly Reddy et al. (2009) explored the potential of certain plant extracts and biocontrol agents for the reduction of aflatoxin B1 (AFB1) in stored rice. Among the plant extracts tested, *Syzygium aromaticum* (L.) Merr. Et Perry, *Curcuma longa* (L.), *Allium sativum* (L.), and *Ocimum sanctum* (Linn.) significantly inhibited the *A. flavus* growth and AFB1 production. Velazhahan et al. (2010) evaluated various medicinal plants extracts for their ability to detoxify aflatoxin G1 (AFG1) by thin-layer chromatography and enzyme-linked immunosorbent assay (ELISA). Of the various plant extracts, the seeds extract of *Trachyspermum ammi* showed maximum degradation of AFG1 after 24 h of incubation. Another study by Vijayanandraj et al. (2014) also demonstrated the effect of different parameters on aqueous extracts of various medicinal plants for detoxification of aflatoxin B1 (AFB1). They concluded that leaf extracts of *Adhatoda vasica* Nees showed the maximum degradation of AFB1 (≥98%) after incubation for 24 h at 37°C. Moreover, Kannan and Velazhahan (2014) explored the potential of some indigenous medicinal plants extracts for detoxification of aflatoxins. The study showed that among several tested plants, *Barleria lupulina* Lindl. leaves extract exhibited maximum detoxification of aflatoxins B1, B2, G1, and G2 at pH 10 whereas detoxification percentage decreased at pH 7 and 3. Time course study of aflatoxin detoxification by *B. lupulina* extract showed that degeneration of aflatoxin occurred within 10 min and this percentage was increased with increase in incubation period.

In current investigation, *Ocimum basilicum* L. (Family: Lamia-ceae, Common name: Sweet basil) and *Cassia fistula* L. (Family: Fabaceae, Common name: Amaltas) were used to evaluate their aflatoxin detoxifying potential. *Ocimum basilicum* is a common herb that is known for its ornamental and therapeutic importance while *C. fistula* is a moderate size deciduous tree, commonly known as golden shower. Both plants have been reported to possess hepatoprotective, antitumor, antitoxic, anti-inflammatory, antibacterial, and antifungal effects. Their Phytochemical studies revealed the presence of terpenoids, alkaloids, flavonoids, tannins, saponin, glycosides, and ascorbic acid (Neelam et al., 2011; Khair-ul-Bariyah et al., 2012). In this present study, these plants were selected to develop a cost effect and environment friendly strategy for aflatoxin detoxification.

## Materials and Methods

### Chemical and Reagents

Aflatoxins B1 and B2 purified from *A. flavus* were prepared in laboratory under optimized conditions and compared through HPLC with standard AFB1 and AFB2 purchased from (Sigma–Aldrich, St. Louis, MO, USA). Stock solutions of AFB1 (1000 μg L^-1^) and AFB2 (500 μg L^-1^) were prepared in methanol and stored at 4°C. The working solutions of AFB1 (100 μg L^-1^) and AFB2 (50 μg L^-1^) were prepared by diluting the stock solution.

### Plant Extract Preparation

*Ocimum basilicum* and *Cassia fistula* samples (leaves and bran-ches) were collected from Quaid-e-Azam campus, University of the Punjab, Lahore. Samples were surface-sterilized using 1% sodium hypochlorite for 10 min and washed several times with sterile distilled water. After surface sterilization, aqueous plants extract were prepared according to the method described by Velazhahan et al. (2010) with some modifications. For extract preparation, 10 g of leaves/branches were homogenize with 10 mL of sterile distilled water. Then homogenate was filtered through muslin cloth and centrifuged at 14,000 rpm for 20 min. Supernatant was sterilized using syringe filter assembly and used for further detoxification studies.

### Fungal Growth Inhibition Assay

Antifungal activity of plants extracts against toxigenic isolates of *A. flavus* was determined by fungal growth inhibition assay as described by Fiori et al. (2000). To 80 ml of malt extract agar medium 20 ml of 10% each filter-sterilized plant extract was added. Then aflatoxigenic isolates of *A. flavus* from 5 days old culture were inoculated centrally into the plates and incubated at room temperature (28 ± 2°C). The colony diameter of test fungi was measured after 4 days by calculating the average radial growth. The percentage inhibition of mycelial growth in relation to the control treatment was calculated by following equation. Three replicates of each treatment were experimented. In control, isolates were inoculated in MEA plates without any plant extract and incubated as described above.

P⁢e⁢r⁢c⁢e⁢n⁢t⁢ g⁢r⁢o⁢w⁢t⁢h⁢ i⁢n⁢h⁢i⁢b⁢i⁢t⁢i⁢o⁢n=((a−b)/a)×100

Where,

*a* = diameter of fungal colony (mean) in control

*b* = diameter of fungal colony (mean) with plant extract

### Detoxification of Aflatoxins (B1 and B2) by Medicinal Plants Extracts (*In Vitro* Studies)

For *In Vitro* detoxification assay, 50 μL of working solution containing (100 μg L^-1^) AFB1 and (50 μg L^-1^) AFB2 was mixed with 250 μL of plants extract and incubated for various intervals of time. Subsequently, the reaction was terminated by adding 250 μL of Chloroform to the above mixture. For recovery of residual toxin mixture was vortex thoroughly. Low speed centrifugation was done for the separation of Chloroform fraction. After that organic phase was withdrawn, evaporated to dryness under gentle stream of nitrogen and redissolved in methanol. Control comprised of toxin: water (50 μL: 250 μL) and incubated under same conditions. All experiments were conducted in triplicate.

### Optimization of Analytical Parameters for Detoxification Assay (*In Vitro*)

#### Temperature and Incubation Time

Plants extracts were incubated with toxins at various intervals of temperature and incubation periods, i.e., 25, 30°C……60°C for 6, 24, and 72 h, respectively. Afterward, the toxin content in the reaction mixture was determined as described above.

#### Optimum pH

The original pH of plants extracts was modified in the range of 2.0–8.0 by using 1 N HCl or 1 N NaoH and then assayed for toxin detoxification activity. Control consists of distilled water with same pH range as well as untreated extract.

### Decontamination of Maize Samples Using Plants Extracts

Maize samples were decontaminated according to method described by Das and Mishra (2000) with some modifications. Ten grams of maize samples were kept in each 250 ml Erlenmeyer flask and spiked with 3 ml of aflatoxins (with concentration B1 100 μg L^-1^ and B2 50 μg L^-1^). These samples were then incubated with 10 ml of aforesaid plants extracts at 30°C for 72 h. After that aflatoxin was extracted according to the modified method of Stroka et al. (2000). Maize samples were incubated with water: acetonitrile (15: 85%) on shaking water bath for 2 h. Afterward the extracts were filtered through filter paper (Whatman, Inc., Clifton, NJ, USA). Then filtrate was passed through afla immunoaffinity column in solid phase extraction assembly. Toxins were slowly eluted from the column with 1 ml of methanol in a glass vial, which was further analyzed by TLC and HPLC. Control comprised of untreated maize sample, sample with plant extract without toxin and sample with toxin without plant extract. Each experiment was performed in triplicate.

### TLC and HPLC Analysis of Aflatoxin

The qualitative analysis of residual toxin was determined by thin layer chromatography (TLC) according to the method described by Ramesh et al. (2013) with some modifications. Twenty-five microliters of methanolic fraction of treated and untreated samples were spotted on 0.25 mm silica gel 60F_254_ (20 cm × 20 cm, Merck). Then plates were developed on chloroform: acetone (92: 8 v/v) and viewed under UV light at 365 nm.

Before quantitative analysis by HPLC, treated and untreated toxins were derivatized by the method described by Hernandez-Hierro et al. (2008) with some modifications. For this purpose, eluted toxin was evaporated to dryness with gentle stream of nitrogen, redissolved in 200 μL *n*-hexane, vortexed and 50 μL of trifluoroacetic acid (TFA) was added in it. Then, 950 μL of acetonitrile–water (1:9) was added to above solution and filtered by using syringe filter assembly. The filtrate was analyzed by HPLC system (Agilent 1100 series, Agilent Technologies, Santa Clara, CA, USA) equipped with a reverse-phase C18 column (Merck, Darmstadt, Germany), and a fluorescence detector. Water:methanol:acetonitrile (60:20:20 v/v/v) were used as mobile phase at a flow rate of 1 mL min^-1.^ Toxins were detected at excitation and emission wavelengths of 360 nm and 440 nm, respectively. For HPLC method validation, calibration curves were drawn using series of calibration solutions in methanol. Each standard solution was chromatographed in duplicate.

### Identification of Degraded Toxin Products

Mass spectral studies were carried out for the identification degraded toxin products.

### LCMS Analysis of Degraded Toxin

Degraded toxin products were analyzed by using surveyor LC system equipped with mass spectrophotometer and PDA plus detectors (Thermo Fisher Scientific). System was validated with known standards individually and in mixture form. All analysis were performed in triplicate using luna phenomenex C_18_ column (150 mm × 4.6 mm, 3 μm), in isocratic mode. Following are the LC-MS conditions for Aflatoxins. Injection volume was 10 μL. Mobile phase consisted of methanol:acetonitrile:water (22.5:22.5:55.0 v/v). Column temperature was maintained at 30°C. The total operation time was 25 min with the flow rate of 0.5 mL min^-1^. MS conditions were as follows: capillary temperature was 335°C, sheath gas flow and auxiliary gas flow was 20 L min^-1^ and 4 L min^-1^, respectively. Source voltage, capillary voltage, and tube lens voltage was 5 KV, 49 V, and 120 V, respectively. Toxins incubated with water under optimized conditions (i.e., temp 30°C and pH 8) were run as a control.

### Electrospray Ionization Mass Spectrometery (ESI – MS/MS) of Aflatoxins through Direct Insertion Pump

In order to predict the molecular formulae as well as elemental composition of AFB1 and AFB2 degradation products, samples were further analyzed by electrospray ionization mass spectrometer. MS/MS was performed on was performed on a Thermo Scientific LTQ XL System fitted with electrospray ionization (ESI) source operating in positive ionization mode with optimum conditions set as follows: capillary voltage to 49.0 V, source voltage to 5.0 KV, Tube lens voltage to 110 V, and capillary temperature to 275°C. Sheath and auxiliary gas flow were adjusted to get stable spray, i.e., 3 L min^-1^ and 0.4 L min^-1^, respectively. Data was collected in positive mode within the range of 100 to 500 m/z. The final identification of an unknown compound was based on the accurate measurement of mass of parent ions and fragments, as well as other useful MS/MS spectrum information (Wang et al., 2011). In MS/MS experiments, untreated aflatoxins and water treated toxins were run as a control.

### Brine Shrimps Bioassay for Toxicity Assessment of Degraded Products

Brine shrimps (*Artemia salina*) bioassay was carried out for testing the biological toxicity of degraded toxin products by the method developed by Solis et al. (1993) with some modifications.

Shrimp eggs (100–200 mg) were hatched in artificial sea water (34 g sea salt/L of deionized water) by incubation under 60 W lamp, providing direct light and warmth (26°C). Throughout hatching period, the same conditions of light sensitivity and temperature were maintained. After an incubation period, the hatched nauplii were separated from shells and transferred to fresh sea water.

Three hundred microliters of treated and untreated AFB1 and AFB2 (100 and 50 μg L^-1^) were added to 96 well plate separately, dried and redissolved in 200 μL of sea water. After that, 200 μL of sea water containing 40–45 organisms were pipetted into each well, resulting in a final volume of 400 μL and incubated for 24–96 h at 26°C. Percentage mortality was determined by counting the immobile (dead) larvae under stereo microscope. Toxicity of each solution was evaluated in triplicate.

### Statistical Analysis

Statistical analysis was carried out by using DSSTAT software. Data were analyzed by analysis of variance (ANOVA) and differences among the means were determined for significance at *P* ≤ 0.05 using Tukey’s multiple range test.

## Results

### Inhibitory Effects of Aqueous Plants Extracts on the Growth of Aflatoxigenic Isolates of *Aspergillus flavus*

*Ocimum basilicum* and *Cassia fistula* aqueous extracts were tested to determine their inhibitory effect on growth of aflatoxigenic isolates of *Aspergillus flavus*. **Table 2** depicted that *O. basilicum* leaves extract showed 82.8–87.7% inhibition of growth of tested isolates. This was followed by *O. basilicum* branch extract with percentage inhibition of 57–68.3% in growth of all tested isolates. While 23.6–49.8% and 16.4–42.3% inhibition in mycelial growth was recorded by aqueous extracts of *C. fistula* leaves and branch, respectively.

### Influence of Temperature and Incubation Period on Toxin Detoxification by Plants Extracts

The aflatoxins (B1 and B2) detoxifying efficacy of *Ocimum basilicum* and *Cassia fistula* (leaves and branches) aqueous extracts were tested at different temperature and incubation time. The degree of detoxification was compared with control experimented under same conditions. Results showed that percentage of detoxification increased with increase in incubation time to 6–72 h (**Table 1**). At lowest tested temperature (25°C), maximum detoxification of AFB1 and AFB2 was shown by *O. basilicum* leaves extract, i.e., 77.9 and 76.7%, respectively, after 72 h of incubation. At this temperature, 36.6–65.7% and 48.9–57.0% reduction in AFB1 and AFB2 level was recorded in samples treated with *C. fistula* leaves and *O. basilicum* branch extract. While least significant detoxification of AFB1 and AFB2 was observed after treatment with *C. fistula* branch extracts, i.e., 30.3 and 43.9%, respectively.

**Table 1:** Effect of temperature and incubation period on aflatoxin detoxification by *O. basilicum* and *C. fistula* aqueous extracts.

Treatments	Temperature	Percentage reduction in AFB1			Percentage reduction in AFB2		
		6 h	24 h	72 h	6 h	24 h	72 h
Toxin	25	0.71^p B^	1.71^m AB^	2.86^o A^	0.53^s BC^	0.84^t B^	1.02^w A^
	30	0.88^p B^	2.30^m AB^	3.81^o A^	0.68^s BC^	0.99^t B^	1.16^w A^
	35	2.54^p C^	3.01^m B^	4.49^o A^	0.82^s B^	1.14^t AB^	1.31^w A^
	40	3.87^p C^	4.33^m B^	5.15^o A^	0.97^s B^	1.29^t AB^	1.46^w A^
	45	5.19^p AB^	5.66^m AB^	5.82^o A^	1.12^s B^	1.44^t AB^	1.61^w A^
	50	6.48^op AB^	6.51^m AB^	6.98^o A^	1.27^s B^	1.59^t AB^	1.76^w A^
	55	7.14^op BC^	7.84^m B^	8.30^o A^	1.42^s B^	1.73^t AB^	1.91^w A^
	60	7.80^op B^	9.16^m AB^	9.63^o A^	1.57^s BC^	1.88^t B^	2.06^w A^
Toxin + water	25	1.48^p B^	3.36^m AB^	3.38^o A^	0.47^s C^	1.47^t B^	2.25^w A^
	30	2.64^p C^	3.56^m B^	4.19^o A^	1.14^s B^	2.04^t AB^	2.41^w A^
	35	3.44^p C^	4.80^m B^	5.45^o A^	1.23^s B^	2.73^t AB^	2.87^w A^
	40	4.76^p A^	6.13^m AB^	6.48^o A^	1.98^s B^	3.84^t AB^	3.40^w A^
	45	6.08^op B^	7.45^m AB^	7.47^o A^	2.72^s C^	3.92^t B^	4.44^w A^
	50	7.41^op B^	8.46^m AB^	8.77^o A^	3.46^s C^	4.43^t B^	6.07^w A^
	55	8.73^op C^	9.46^m B^	10.10^o A^	4.21^s AB^	4.96^t B^	7.19^w A^
	60	10.05^op BC^	10.45^m B^	11.42^o A^	4.95^s C^	5.48^t B^	8.31^w A^
T + *C. fistula* leaves	25	23.81^lmn C^	30.00^kl B^	36.65^lmn A^	32.20^pqr C^	41.13^p–s B^	48.94^r–u A^
	30	27.68^k–n C^	30.74^jkl B^	37.84^k–n A^	33.94^opq C^	43.36^o–r B^	50.80^p–t A^
	35	32.15^j–n C^	34.32^i–l B^	40.52^j–n A^	36.17^nop C^	44.85^m–r B^	52.29^o–s A^
	40	39.59^h–k C^	44.74^f–I B^	47.67^h–l A^	38.40^mno C^	46.34^k–q B^	53.78^n–q A^
	45	41.99^g–k C^	45.93^f–I B^	51.24^hij A^	40.64^lmn C^	47.83^j–p B^	55.27^mno A^
	50	43.16^f–j C^	46.82^f–I B^	52.43^hi A^	42.87^klm C^	49.32^i–o B^	56.76^lmn A^
	55	45.25^d–j C^	50.10^e–h B^	52.73^hi A^	45.10^jkl C^	50.80^h–n B^	58.24^klm A^
	60	46.74^d–j C^	50.69^e–h B^	53.92^h A^	47.33^ijk C^	52.29^g–l B^	59.73^jkl A^
T + *C. fistula* branch	25	19.87^no C^	25.06^l B^	30.30^n A^	27.24^r C^	36.17^s B^	43.98^v A^
	30	23.15^mn C^	25.80^l B^	31.49^n A^	28.98^qr C^	38.40^rs B^	45.84^uv A^
	35	27.62^k–n C^	29.38^kl B^	34.17^mn A^	31.21^pqr C^	39.89^qrs B^	47.33^tuv A^
	40	35.06^j–m C^	39.80^h–k B^	41.31^i–n A^	33.44^opq C^	41.38^p–s B^	48.82^stu A^
	45	36.67^i–m C^	40.99^g–k B^	44.89^h–m A^	35.67^nop C^	42.87^o–s B^	50.31^q–t A^
	50	38.04^i–l C^	41.88^g–k B^	46.08^h–l A^	37.90^mno C^	44.35^n–r B^	51.79^o–s A^
	55	40.12^g–k C^	45.16^f–I B^	47.87^h–l A^	40.14^lmn C^	45.84^l–q B^	53.28^n–r A^
	60	41.61^g–k C^	45.75^f–I B^	49.06^h–k A^	42.37^klm C^	47.33^k–p B^	54.77^m–p A^
T + *O. basilicum* leaves	25	54.41^c–g C^	66.62^bcd B^	77.91^de A^	70.08^f C^	74.55^e B^	76.78^f A^
	30	58.29^b–e C^	67.37^bcd B^	79.10^cde A^	71.82^ef C^	76.78^de B^	79.01^ef A^
	35	62.75^abc C^	70.94^abc B^	81.78^a–d A^	74.05^def C^	78.27^cde B^	80.50^def A^
	40	69.0^ab C^	78.09^ab B^	86.54^a–d A^	76.28^cde C^	79.76^b–e B^	81.99^cde A^
	45	72.64^a C^	79.28^ab B^	90.12^abc A^	78.52^bcd C^	81.99^a–d B^	83.48^bcd A^
	50	73.47^a C^	79.57^ab B^	91.31^ab A^	80.75^abc C^	83.48^abc B^	84.96^abc A^
	55	74.81^a C^	82.85^a B^	91.60^ab A^	82.98^ab C^	84.96^ab B^	86.45^ab A^
	60	76.30^a C^	83.45^a B^	92.80^a A^	85.21^a B^	87.20^a AB^	87.94^a A^
T + *O. basilicum* branch	25	35.42^j–m C^	43.70^f–j B^	65.73^g A^	40.32^lmn C^	49.25^i–o B^	57.06^lmn A^
	30	39.29^ijk C^	44.44^f–I B^	66.93^fg A^	42.06^klm C^	51.48^h–m B^	58.92^klm A^
	35	43.76^d–j C^	48.02^e–I B^	69.61^efg A^	44.29^jkl C^	52.97^g–k B^	60.41^jkl A^
	40	51.20^c–I C^	55.16^d–g B^	76.75^def A^	46.52^ijk C^	54.46^f–j B^	61.90^ijk A^
	45	54.03^c–h C^	56.36^def B^	80.32^b–e A^	48.75^hij C^	55.95^f–I B^	63.39^hij A^
	50	54.78^c–g C^	57.25^def B^	81.52^a–d A^	50.99^ghi C^	57.43^fgh B^	64.87^ghi A^
	55	56.86^b–f C^	60.52^cde B^	81.80^a–d A^	53.22^gh C^	58.92^fg B^	66.36^gh A^
	60	58.35^bcd C^	61.12^cde B^	83.00^a–d A^	55.45^g C^	60.41^f B^	67.85^g A^

*Values are mean of three replicates. Small alphabetic letters with different values indicate significant differences (*p* < 0.05) in toxin reduction at different temperature and incubation periods in each column. Capital alphabetic letters with different values indicate significant differences (*p* < 0.05) among controls and tested plant extracts at different temperature and incubation period in each row*.

**Table 2:** Effects of aqueous plants extracts on mycelial growth inhibition of *Aspergillus flavus* isolates.

Aqueous plant extracts	Percentage of mycelial growth inhibition of *Aspergillus flavus* isolates				
	Isolate 24	Isolate 31	Isolate 37	Isolate 44	Isolate 42


*C. fistula* branch	35.4^d^	42.3^cd^	16.4^d^	32.8^cd^	39.3^cd^


*C. fistula* leaves	41.5^c^	49.8^c^	23.6^c^	37.4^c^	44.8^c^


*O. basilicum* branch	67.7^b^	65.2^b^	57.0^b^	64.9^b^	68.3^b^


*O. basilicum* leaves	87.7^a^	87.6^a^	84.8^a^	82.8^a^	87.4^a^

*Values are mean of three replicates. Values with different alphabetic letters indicate significant differences among tested plants extract as determined by Tukey’s multiple range test*.

Similarly, at 30°C most significant detoxification of AFB1 and AFB2 was shown by *O. basilicum* leaves extract which was followed by *O. basilicum* branch, *C. fistula* leaves and branch extracts, after 72 h of incubation. In control samples at 30°C only 4.19 and 2.41% AFB1 and AFB2 were found to be inactivated (**Table 1**).

The findings of this study depicted that the percentage detoxification of AFB1 and AFB2 by plants extracts was gradually increased with the consequential increase in temperature to 35–55°C. Similarly highest detoxification of AFB1 and AFB2 was observed at 60°C but this detoxification may be due to synergistic effect of heat and moisture. In present study, *O. basilicum* leaves extracts showed highest detoxification of aflatoxins at all tested temperatures and incubation periods. However, further experimentations were carried out at 30°C because it is more or less near to room temperature and also found to be prevailing temperature in most of the storehouses of Punjab especially in summer, which may provide a cost effective approach to detoxify these toxins.

### Effect of pH on Detoxification of Aflatoxins by Plants Extracts

This study was aimed to observe the detoxifying effect of pH using *O. basilicum* and *C. fistula* aqueous extracts. Distilled water with pH adjusted to 2, 4, 6, and 8 was used as a control. Data revealed that least significant detoxification was observed at pH 2. At this pH, maximum detoxification AFB1 and AFB2 was observed in samples treated with *O. basilicum* leaves extract, i.e., 78.8 and 72.7%, respectively (**Table 3**). This was followed by *O. basilicum* branch, *C. fistula* leaves and branch extracts.

**Table 3:** Effect of pH on detoxification of AFB1 and AFB2 by *C. fistula* and *O. basilicum* aqueous extracts after 72 h of Incubation.

Treatments	% Detoxification AFB1				% Detoxification AFB2			
	pH 2	pH 4	pH 6	pH 8	pH 2	pH 4	pH 6	pH 8


Toxin + water	12.67^c C^	13.88^d C^	14.87^d B^	16.49^d A^	5.43^d D^	9.69^d C^	11.00^d B^	12.86^d A^


*C. fistula* leaves + toxin	40.12^bc C^	39.23^c BC^	40.58^c B^	54.37^c A^	36.68^c D^	48.67^c C^	51.03^bc B^	53.05^c A^


*C. fistula* branch + toxin	34.83^bc C^	35.48^c BC^	36.85^c B^	40.73^c A^	32.95^c D^	44.35^c C^	47.25^c B^	49.47^c A^


*O. basilicum* leaves + toxin	78.81^a D^	80.62^a C^	83.58^a B^	90.43^a A^	72.77^a C^	76.08^a BC^	77.37^a B^	88.69^a A^


*O. basilicum* branch + toxin	46.67^b D^	52.34^b C^	63.45^b B^	74.97^b^	44.72^b D^	55.86^b C^	59.50^b B^	62.57^b A^

*Values are mean of three replicates. Small alphabetic letters with different values indicate significant differences (*p* < 0.05) among tested plant extracts. Capital alphabetic letters with different values indicate significant differences (*p* < 0.05) among pH treatments*.

The percentage of detoxification increases with increase in pH from acidic to alkaline range. Hence, the highest degradation of AFB1 and AFB2 was recorded at pH 8 after treatment with *O. basilicum* leaves extracts, i.e., 90.4 and 88.6%, respectively. While 40.7–74.9 and 49.4–62.5% detoxification of AFB1 and AFB2 was shown by *O. basilicum* branch, *C. fistula* leaves and branch extracts, respectively. At this pH respective control showed 16.4 and 12.8% degradation of AFB1 and AFB2 (**Table 3**). So, further studies were done at pH 8 as the results clearly depicted that the efficacy of plants extracts to detoxify AFB1 and AFB2 significantly enhanced at this pH.

### Decontamination of Maize Samples by Plants Extracts

This study was conducted under optimized condition of *In Vitro* assays, i.e., pH (8), temp (30°C) and incubation time (72 h). According to results, only 0.5–0.3 μg L^-1^ AFB1 and AFB2 were found to be present in unspiked control maize samples. However, 97.3 μg L^-1^ of AFB1 and 47.6 μg L^-1^ of AFB2 were recovered from control maize samples spiked with 100 μg L^-1^ AFB1 and 50 μg L^-1^ AFB2 (**Table 4**).

**Table 4:** Decontamination of maize samples by *C. fistula* and *O. basilicum* aqueous extracts under optimized conditions.

	Toxin recovery μg L^-^^1^ (% detoxification)	
	AFB1	AFB2
**Control**		
Unspiked maize	0.5	0.3
Unspiked maize + *C. fistula* leaves extract	0.0	0.0
Unspiked maize + *C. fistula* branch extract	0.0	0.0
Unspiked maize + *O. basilicum* leaves extract	0.0	0.0
Unspiked maize + *O. basilicum* branch extract	0.0	0.0
Spiked maize sample (AFB1: 100 μg L^-1^; AFB2: 50 μg L^-1^)	97.3	47.6
**Treatment**		
Maize + *C. fistula* leaves extract	56.9 (43.1^c^)	25.2 (49.6^c^)
Maize + *C. fistula* branch extract	62.3 (37.7^d^)	28.6 (42.7^d^)
Maize + *O. basilicum* leaves extract	13.1 (86.9^a^)	8.3 (83.5^a^)
Maize + *O. basilicum* branch extract	32.7 (67.3^b^)	21.9 (56.1^b^)

*Values are mean of three replicates. Figures in the parenthesis are detoxification % age. Values with different alphabetic letters indicate significant differences (*P* < 0.05) as determined by Tukey’s multiple range test*.

The findings of this study depicted a similar detoxifying trend as that was recorded in *In Vitro* studies. Results showed that aqueous extracts of *O. basilicum* leaves found as most significant source to detoxify AFB1 and AFB2 as they showed detoxification percentage of 86.9 and 83.5%, respectively in spiked maize samples. While in spiked samples, 37.7–67.3% and 42.7–56.1% detoxification of AFB1 and AFB2 was recoded after treatment with *O. basilicum* branch, *C. fistula* leaves and branch extracts (**Table 4**).

In this present investigation, overall results of various assays demonstrated that *O. basilicum* leaves extracts degraded AFB1 and AFB2 most significantly under optimized conditions as compared to *O. basilicum* branch, *C. fistula* leaves and branch extracts.

**FIGURE 1:**
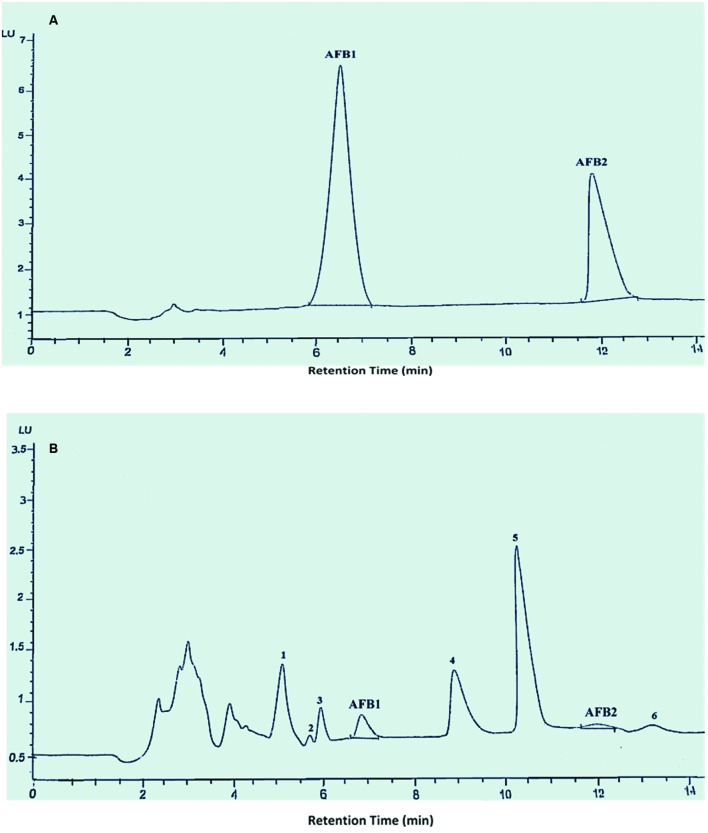
**HPLC chromatogram of AFB1 and AFB2**. Whereas **(A)** untreated toxins; **(B)** toxin treated with *O. basilicum* leaves extract at 30°C and pH 8.

Moreover HPLC chromatograms that obtained after treat-ment with *O. basilicum* leaves extracts, showed the presence of various peaks whose footprints were not found in chromatogram of control which may be attributed to toxin degradation products (**Figure 1**). So, aflatoxins degraded with *O. basilicum* leaves extracts were used in further structural characterization studies for the identification of degradation products.

### Structural Characterization of Aflatoxin and Their Degradation Products

Aflatoxins showed good ESI ionization in positive ion mode. A molecular base ion at m/z 313.17 and m/z 315.17 was detected for protonated adduct [M+ H]^+^ of aflatoxins B1 and B2 while sodium adduct [M+ Na]^+^ was formed at m/z 335 and m/z 337, respectively. The identity of parent ion was validated by its fragmentation into daughter ions. The protonated molecule was chosen as the precursor ion for aflatoxins in the product ion scan mode because the sodium adduct did not exhibit specific fragmentation for any compound. The fragmentation pattern of AFB1 and AFB2 has an important reference value in analyzing fragmentation pathway of degraded products.

### MS/MS Analysis of AFB1 and AFB2

**FIGURE 2:**
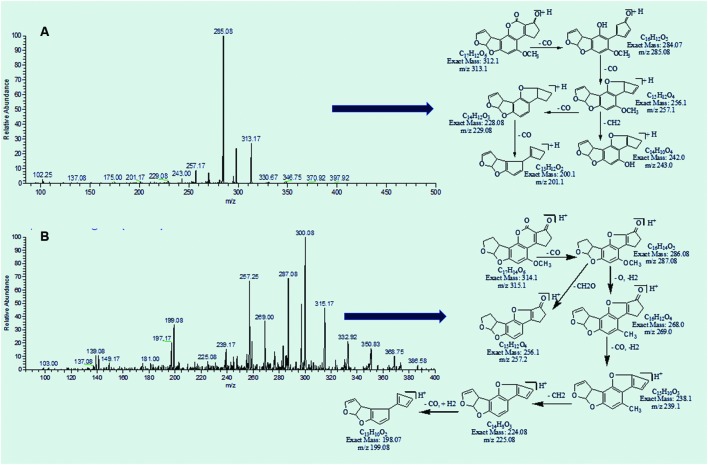
**MS/MS Spectra and fragmentation pathway. (A)** AFB1 and **(B)** AFB2.

AFB1 is composed of 17 carbon atoms, 6 oxygen, and 12 hydrogen atoms while AFB2 contains 14 hydrogen atoms in their formulae along with 17 carbon and 6 oxygen atoms. MS/MS spectrum revealed that continuous loss of carbon monoxide (CO) was the main fragmentation pathway of AFB1. Methyl and methanol losses occurred on methoxy group located on side chain of benzene. The double bond equivalence (DBE) of AFB1 was 12 (**Figure 2A**). However, MS/MS fragmentation pathway of AFB2 illustrated that daughter ions were formed by loss of carbon monoxide, oxygen, hydrogen, and methyl group (**Figure 2B**). The DBE of AFB2 was 11. Identification of degradation products were based on accurate mass measurement of ions and similar fragmentation pathways with that of AFB1 and AFB2.

**FIGURE 3:**
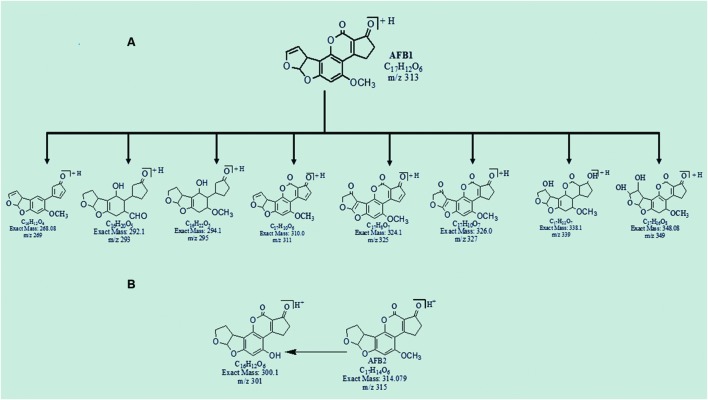
**Possible degraded products of **(A)** AFB1 and **(B)** AFB2 after treatment with *O. basilicum* leaves extracts at 30°C and pH 8**.

It is evidenced from the results that nine degraded products were obtained after detoxification of AFB1 and AFB2 by using *O. basilicum* leaves extract. Among them, some were formed by the modification of lactone ring while others were produced as a result of addition reaction in furan rings of aflatoxins. Structural formulas of possible degraded products of AFB1 and AFB2 are shown in **Figures 3A,B**.

### MS/MS Analysis of Degraded Products of AFB1

**FIGURE 4:**
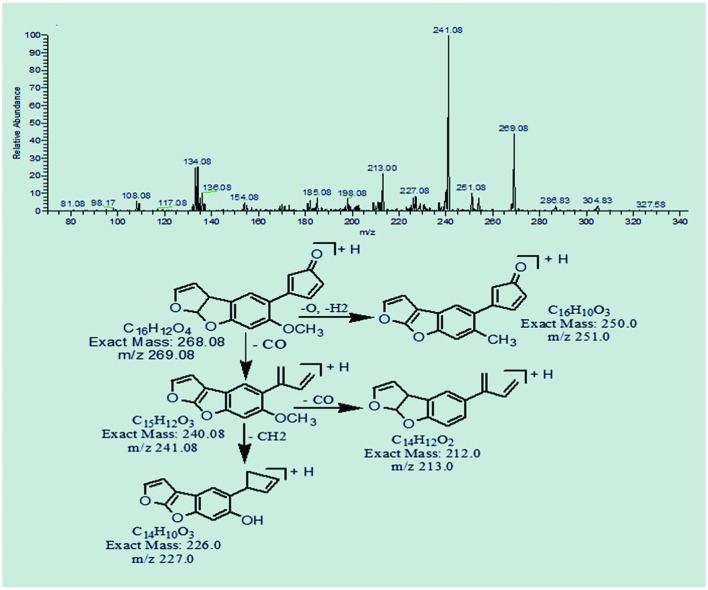
**MS/MS spectra and fragmentation pathway of degradation product with 269.08 m/z**.

The degradation product at m/z 269.08 corresponded to molecular formula C_16_H_12_O_4_ formed by the loss of carbon dioxide from lactone ring of AFB1. The DBE of C_16_H_12_O_4_ was one less than AFB1. Loss of oxygen, methyl group and carbon monoxide was the main fragmentation pathway (**Figure 4**).

**FIGURE 5:**
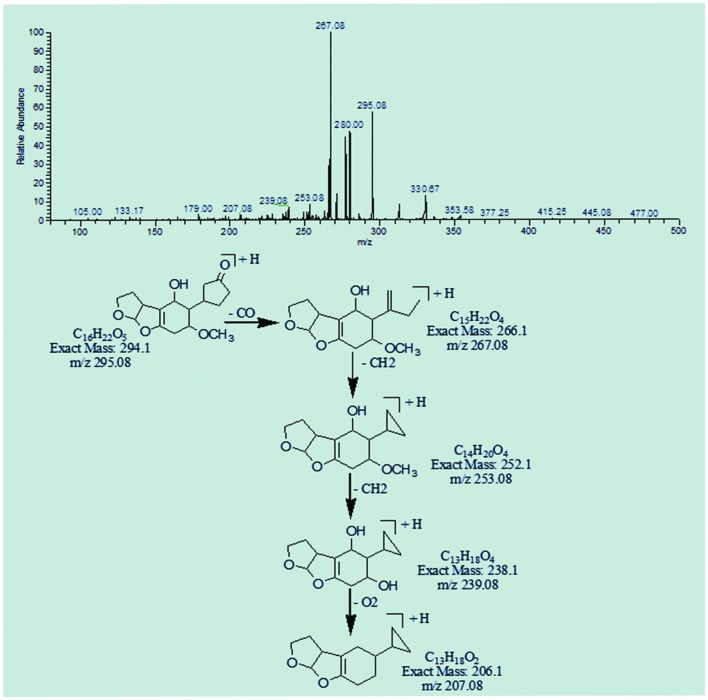
**MS/MS spectra and fragmentation pathway of degradation product with 295.08 m/z**.

**FIGURE 6:**
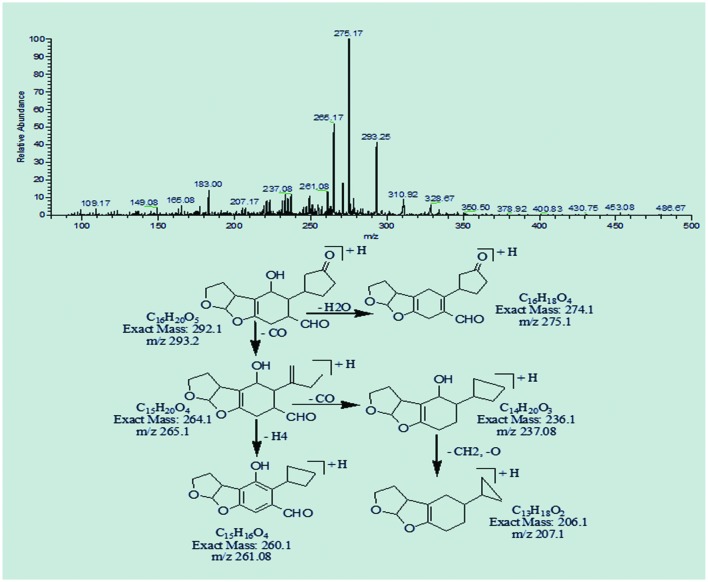
**MS/MS spectra and fragmentation pathway of degradation product with 293.25 m/z**.

The degradation products C_16_H_22_O_5_ (m/z 295.08) and C_16_H_20_O_5_ (m/z 293.25) were formed due to the loss of carbon monoxide by the opening of lactone ring and addition of hydrogen atom to AFB1 molecule. The DBE content of both products was same, i.e., six while difference between them was of two hydrogen atoms. Losses of CO, CH_2_, and O were the main fragmentation pathway of both products (**Figures 5** and **6**).

**FIGURE 7:**
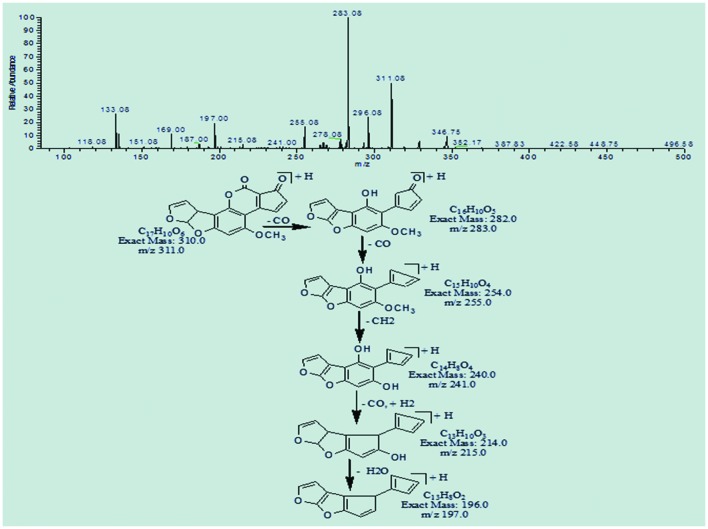
**MS/MS spectra and fragmentation pathway of degradation product with 311.08 m/z**.

Degradation product C_17_H_10_O_6_ with ion peak at m/z 311.08 was formed by the loss of hydrogen atoms implying greater DBE content than that of AFB1. The fragmentation pathway of C_17_H_10_O_6_ was different from that of AFB1. The precursor ion yielded a series of product ions represented by 283.08[M-CO]^+^, 255.08[M-C_2_O_2_]^+^, 241.0[M-C_3_H_2_O_2_]^+^, 215.08[M-C_4_O_3_]^+^, and 197.0[M-C_4_H_2_O_4_]^+^ (**Figure 7**).

**FIGURE 8:**
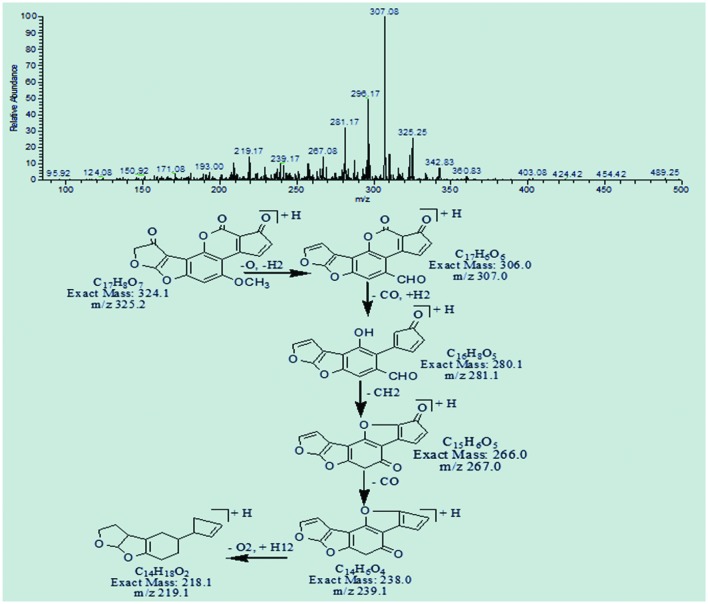
**MS/MS spectra and fragmentation pathway of degradation product with 325.25 m/z**.

**FIGURE 9:**
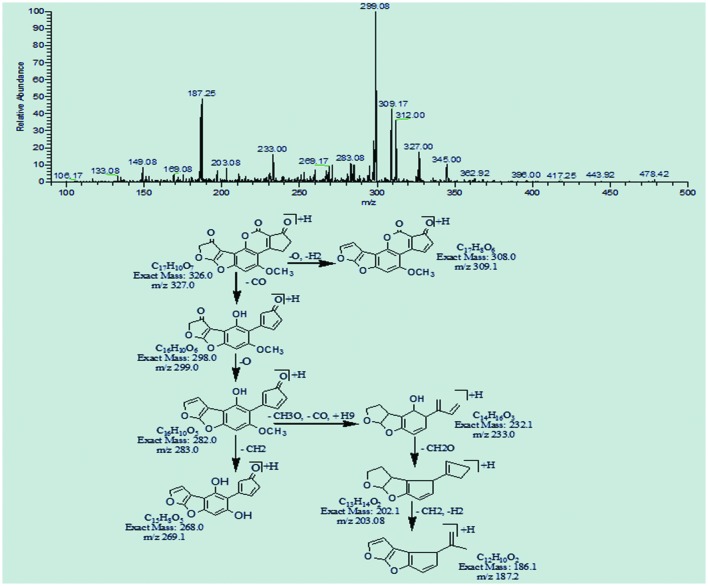
**MS/MS spectra and fragmentation pathway of degradation product with 327.0 m/z**.

The degradation products C_17_H_8_O_7_ (with m/z 325.25) and C_17_H_10_O_7_ (with m/z 327.0) were formed by the addition of oxygen atom on the double bond of terminal furan ring on the left side. The difference between these two products was only of two hydrogen atoms. The DBE content of both products was greater than AFB1, i.e., 14 and 13, respectively. The precursor ion C_17_H_8_O_7_ yielded a series of product ions represented by 307.08[M-H_2_O]^+^, 281.17[M-CO_2_]^+^, 267.08[M-C_2_H_2_O_2_]^+^, and 239.17[M-C_3_H_2_O_3_]^+^. While the fragmentation pathway of C_17_H_10_O_7_ showed losses of O, CO, CH_2_, and CH_3_O groups (**Figures 8** and **9**).

**FIGURE 10:**
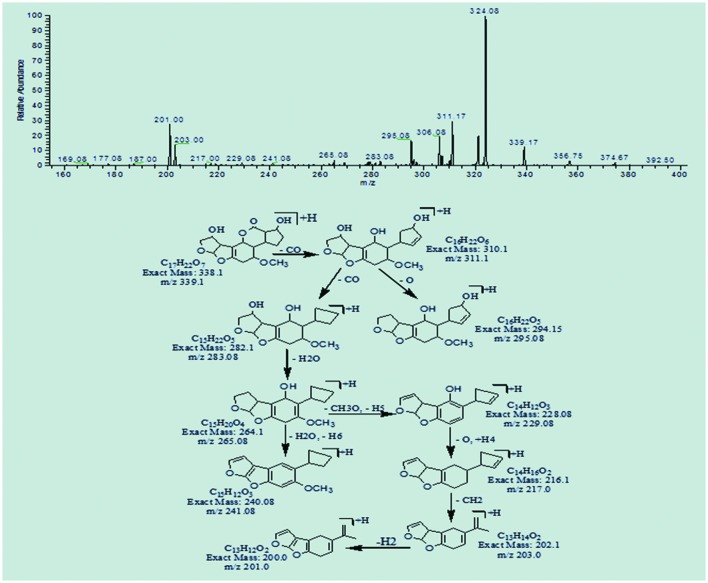
**MS/MS spectra and fragmentation pathway of degradation product with 339.1 m/z**.

The degradation product C_17_H_22_O_7_ at m/z 339.17 produced as a result of addition reaction on furan rings. Double bonds were replaced by the addition of hydrogen atoms with lower DBE content than that of AFB1 i.e., seven. The fragmentation pathway was different from that of AFB1. Fragments of C_17_H_22_O_7_ showed losses of carbon monoxide, water, oxygen, and methyl groups. More details on fragmentation pathway are shown in **Figure 10**.

**FIGURE 11:**
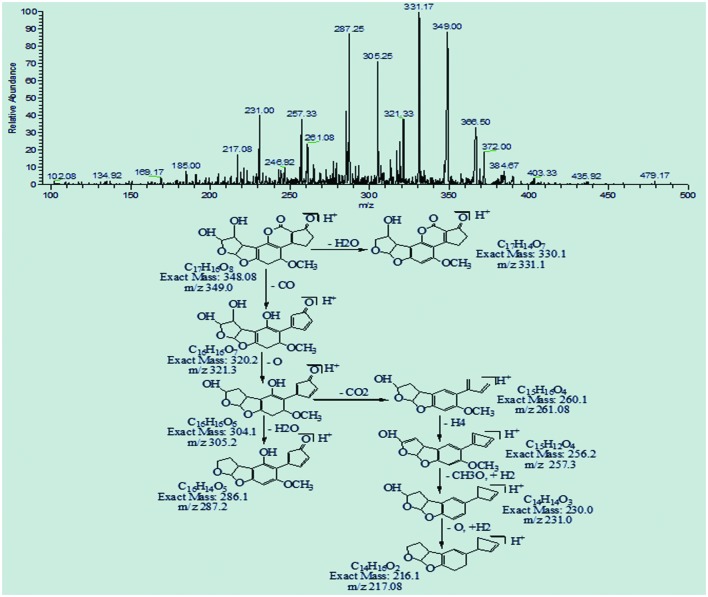
**MS/MS spectra and fragmentation pathway of degradation product with 349.0 m/z**.

The degradation product C_17_H_16_O_8_ (with 349.0 m/z) had one more H_4_O_2_ molecule and less DBE content than AFB1, i.e., 10. The result is most likely caused by additional reaction of two hydroxyl groups on the double bond of terminal furan ring. The precursor ion yielded a series of product ions represented by 331.17[M-H_2_O]^+^, 321.33[M-CO]^+^, 305.25[M-CO_2_]^+^, 287.25[M-CH_2_O_3_]^+^, 261.08[M-C_2_O_4_]^+^, 257.33[M-C_2_H_4_O_4_]^+^, 231.00 [M-C_3_H_2_O_5_]^+^, and 217.08[M-C_3_O_6_]^+^ (**Figure 11**).

### MS/MS Analysis of AFB2 Degradation Product

**FIGURE 12:**
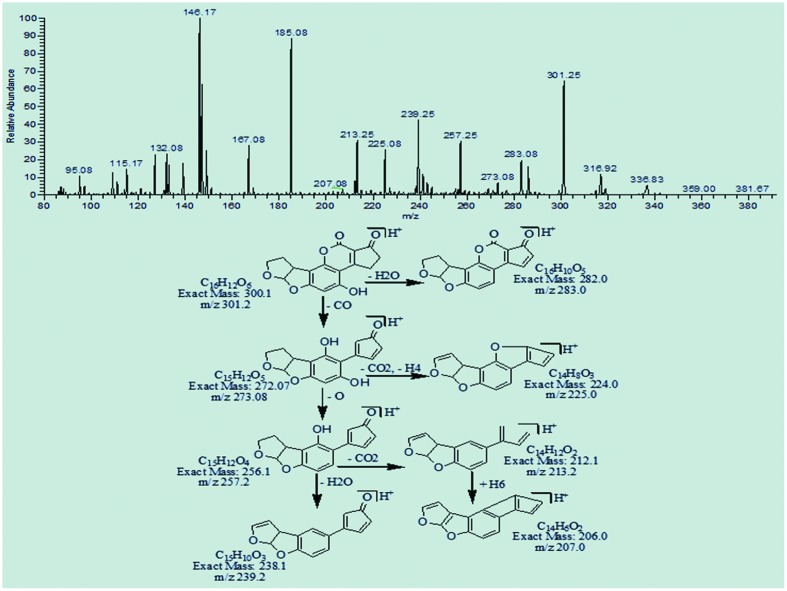
**MS/MS spectra and fragmentation pathway of degradation product with 301.2 m/z**.

The degradation product at m/z 301.25 with molecular formula C_16_H_12_O_6_ was formed by the replacement of methoxy group with hydroxyl group on the side chain of benzene ring. The DBE of C_16_H_12_O_6_ was same as that of AFB2. Fragmentation pathway involves the loss of carbon dioxide, carbon monoxide, and water molecules (**Figure 12**).

### Estimation of Toxicity of Degradation Products

The biological toxicity of untreated and treated aflatoxins B1 and B2 were compared through brine shrimps (*Artemia salina*) bioassay to confirm the safety of degraded products. Hatched shrimps larvae were incubated with treated and untreated aflatoxins at 26°C for 24–96 h to observe the mortality response of larvae. Natural mortalities were determined in blank sea water and in well treated with solvent only. The percentage of mortality was calculated from average number of dead larvae per concentration.

Larvae incubated with untreated aflatoxins showed significant increase in larval mortality as compared to control, i.e., 83%. This percentage of mortality was increased to 91.7% with increase in incubation time. As compared to untreated toxins, significant reduction in larval mortality was recorded after incubation with treated toxins. Data derived from tests conducted with AFB1 (100 μg L^-1^) and AFB2 (50 μg L^-1^) degraded by *O. basilicum* leaves extracts showed only 9.2–22.5% mortality of shrimps larvae after 96 h of incubation (**Table 5**). The outcome of this study clearly implies that aflatoxins degradation products showed much lower toxicity toward brine shrimps larvae than untreated aflatoxins

**Table 5:** Effect of detoxified toxins on percent mortality of Brine shrimp larvae at 26°C after 24–96 h after incubation.

Treatments	Incubation period (h)	No. of living shrimps	No. of dead shrimps	% Mortality
**Control**				
Sea water + shrimps	24	40	0	0


	48	40	0	0
	72	40	0	0
	96	39	1	2.5
Methanol + shrimps	24	38	1	2.5
	48	38	2	5
	72	37	3	7.5
	96	36	3	7.5
Untreated toxins + shrimps	24	7	33	83.0


	48	5	35	86.7
	72	4	36	89.2
	96	3	37	91.7
**Treatment**				
Treated toxin with *O. basilicum* leaves extract + shrimps	24	36	4	9.2


	48	35	5	13.3
	72	32	8	20.0
	96	31	9	22.5

*Values are mean of three replicates. Data was analyzed by analysis of variance (ANOVA)*.

## Discussion

Use of certain plant extracts and biocontrol agents as a source of safer and more effective control on the growth of aflatoxigenic fungi and aflatoxin production have been under investigation by many authors (Gowda et al., 2004; Joseph et al., 2005; Suleiman et al., 2008; Reddy et al., 2009). Much emphasis was given to herbal, medicinal, and aromatic plants for their antifungal activities against food spoilage and aflatoxigenic fungi (Soliman and Badeaa, 2002; Maraqa et al., 2007; Gandomi et al., 2009; El-Nagerabi et al., 2012). Plants are rich source of bioactive secondary metabolites such as tannins, terpenoids, alkaloids, and flavonoids, reported to have antifungal properties (Nwachukwu and Umchuruba, 2001).

In the present study, aqueous extracts of *O. basilicum* and *Cassia fistula* were tested for their antifungal potential against aflatoxigenic isolates of *A. flavus*. All tested plants extracts exhibited diverse degree of antifungal activity against *A. flavus* isolates. The maximum antifungal activity was shown by aqueous extract of *O. basilicum* leaves followed by *O. basilicum* branch, *C. fistula* leaves and branch extract. Similarly a study conducted by Tanackov et al. (2011) also showed that extracts of *O. basilicum* significantly inhibit the growth of the *Fusarium oxysporum*, *F. proliferatum*, *F. subglutinans*, and *F. verticillioides*. Antifungal potential of aforementioned plants used in this study, were also described by many scientists (Abo et al., 1999; Phongpaichit et al., 2004; Pujo et al., 2009; Bhalodia and Shukla, 2011).

According to previously documented literature, essential oils and extracts of various spices and herbs like cinnamon, peppermint, basil, and lemongrass can be suggested as plant based safe food additive in protecting food and feed from fungal and mycotoxin contamination (Montes-Belmont and Carvajall, 1998; Burt, 2004; Yang et al., 2007). In this study, *In Vitro* assays were performed with *O. basilicum* and *Cassia fistula* aqueous extracts under optimized conditions of temperature, pH and incubation time. In case of temperature, highest detoxification percentage was observed at 60°C but this detoxification could be due to synergistic action of heat and moisture (Basappa and Shantha, 1996; Rustom, 1997). Similar findings were recorded in a study conducted by Hajare et al. (2005) who worked on aflatoxin inactivation by using Ajwain seeds extract under optimized conditions. Their results showed that highest inactivation was obtained at 60°C but further studies were conducted on 45°C to eliminate the effect of heat and moisture on toxin inactivation. Likewise in this present investigation 30°C was selected for further studies.

The results of present study depicted that percentage of detoxification increase with increase in incubation time to 72 h. These observations are in close agreement to the findings of earlier workers (Hajare et al., 2005; Velazhahan et al., 2010; Kannan and Velazhahan, 2014; Vijayanandraj et al., 2014). Furthermore in *In Vitro* assays, pH of reaction mixture was optimized and found that percentage of detoxification increases as the pH change from acidic to alkaline range. In this study highest detoxification of AFB1 and AFB2 was shown by *O. basilicum* leaves extract at pH 8. Similar finding were obtained by Kannan and Velazhahan (2014) who explored the potential of *Barleria lupulina* leaf extract on detoxification of aflatoxins. In addition, Méndez-Albores et al. (2004) found that aflatoxin florescence, attributed to the coumarin moiety, diminish or even disappear in alkaline treatment.

In the past, numerous researchers observe the structural changes in aflatoxin molecule after detoxification by various means, i.e., micro-organisms, physical and chemical agents, ultraviolet (UV) rays, Gamma rays, and plant products (Alberts et al., 2006; Albores et al., 2008; Guan et al., 2010; Velazhahan et al., 2010; Wang et al., 2011; Farzaneh et al., 2012; Inoue et al., 2013; Luo et al., 2013; Samuel et al., 2014; Vijayanandraj et al., 2014). Lee et al. (1981) observed that lactone ring plays an important role in fluorescence of aflatoxin molecule. On its cleavage, the molecule becomes non-florescent with subsequent significant reduction in toxicity. The toxicity of aflatoxins has been studied by various scientists (Guengerich, 2001, 2008; Hussein and Brasel, 2001). Their toxicity data demonstrated that aflatoxins have cyclopentene ring and furan moiety in their chemical structure. In AFB1 presence of double bond in the terminal furan ring is key factor for its toxic and carcinogenic activities (Wang et al., 2011). As compare to AFB1, the toxicity of AFB2 is hundreds times less due to presence of saturated furan ring (Dvorackova, 1990). The degraded products of AFB2 may be active but were less potent than that of parent compound. Thus removing the double bond of terminal furan ring and modification of lactone ring are major aims of detoxification.

In this present study, data recovered from HPLC revealed that after treatment with *O. basilicum* leaves extracts AFB1 and AFB2 were degraded into number of other compounds whose properties are different from parent toxins. So, the presence of these degraded products was further confirmed by LCMS/MS studies. It is evidenced from the results that eight degraded products were obtained after detoxification of AFB1 by using *O. basilicum* leaves extract. Among them, 50% of degraded products (with m/z 339, 325, 349, and 327) were formed by removal of double bond in furan ring whereas in 25% of products obtained at m/z 293 and 295 modification of lactone ring and removal of double bond in furan ring was occurred. Other studies in literature also supported the similar findings. Velazhahan et al. (2010) reported detoxification of aflatoxin G1 by seed extract of Ajowan (*T. ammi*) and suggested the modification of lactone ring structure of AFG1 as mechanism of detoxification. Similar findings were observed by Vijayanandraj et al. (2014) after detoxification of aflatoxin B1 by an aqueous extract from leaves of *Adhatoda vasica* Nees. A study conducted by Wang et al. (2011) on structure elucidation of radiolytic products of aflatoxin B1 in methanol water solution revealed that in most of radiolytic products addition reaction occurred on the double bond of terminal furan ring, resulting significantly reduced toxicity as compared to that of AFB1. Experiments by Luo et al. (2013) showed that aflatoxin B1 can be effectively degraded by using ozone in aqueous system. According to them, due to conjugate addition reaction on the double bond of terminal furan ring for AFB1, the toxicity of degradation products was significantly decreased compared with that of AFB1.

Brine shrimps (*Artemia salina*) larvae appears to be as susceptible as biological indicator of toxicity of some mycotoxins in foods and feeds (Hartl and Humpf, 2000; Favilla et al., 2006). Several studies in the past were conducted on brine shrimps larvae to evaluate the toxic effects of aflatoxins (Harwing and Scott, 1971; Schmidt, 1989; Logrieco et al., 1996; Durakovic et al., 2002; Moretti et al., 2007). In this study hatched shrimps larvae were incubated with treated and untreated aflatoxins at 26°C for 24 to 96hrs to observe the mortality response of larvae. As compared to untreated toxins, significant reduction in larval mortality was recorded after incubation with treated toxins. Therefore, toxicity of most of degraded products compared with that of aflatoxin was reduced to a much lower level. The reason behind this is that most of the degradation products obtained after treatment with *O. basilicum* leaves extracts were formed as a result of addition reaction on the double bond of terminal furan ring and modification of lactone ring as indicated by mass spectral analysis which is most significant determinant of toxic and carcinogenic activities of aflatoxins. These results were in close agreement with those of Samuel et al. (2014) who worked on detoxification of aflatoxin B1 by *Pseudomonas putida*. He compared the toxicity of treated and untreated AFB1 toward HeLa cells and concluded that degraded products are non-toxic (D1) or much less toxic (D2 and D3) than AFB1 to the cells at the tested concentrations.

## Conclusion

The efficient detoxification of aflatoxins by using *O. basilicum* leaves extracts under optimized conditions. These extracts are easily available, cost effective, biologically safe and provide an excellent alternative source of toxin detoxification instead of physical and chemical methods. Direct spray of aqueous plant extract is easy to prepare and convenient for the farmers as no technical knowledge is involved but the formulations may vary and cause errors in treatment. This trait should be properly investigated. Another most important aspect is the shelf life of the plant extract preparations that should sustain variations in environmental conditions and remain feasible for the effective detoxification of aflatoxins. Future implications of this approach are quite promising in order to overcome world food hunger improving the quality of food items.

## Author Contributions

TA is the supervisor and MA is co-supervisor of this work. WI has worked as research scholar for this project. AG and MI has provided technical guidance for various analyses. AMK has helped in data interpretation.

## Conflict of Interest Statement

The authors declare that the research was conducted in the absence of any commercial or financial relationships that could be construed as a potential conflict of interest.
